# Cortisol directly impacts *Flavobacterium columnare* in vitro growth characteristics

**DOI:** 10.1186/s13567-016-0370-9

**Published:** 2016-08-17

**Authors:** Annelies Maria Declercq, Johan Aerts, Bart Ampe, Freddy Haesebrouck, Sarah De Saeger, Annemie Decostere

**Affiliations:** 1 Department of Pathology, Bacteriology and Poultry Diseases, Faculty of Veterinary Medicine, Ghent University, Ghent, Belgium; 2 Stress Physiology Research Group, Laboratory of Food Analysis, Department of Bio-analysis, Faculty of Pharmaceutical Sciences, Ghent University, Ghent, Belgium; 3 Stress Physiology Research Group, Animal Sciences Unit, Institute for Agriculture and Fisheries Research, Melle, Belgium; 4 Biostatistics and Data modeling, Animal Sciences Unit, Institute for Agriculture and Fisheries Research, Melle, Belgium; 5 Laboratory of Food Analysis, Department of Bio-analysis, Faculty of Pharmaceutical Sciences, Ghent University, Ghent, Belgium

## Abstract

Teleost fish faced with stressful stimuli launch an endocrine stress response through activation of the hypothalamic-pituitary-interrenal axis to release glucocorticoids, in particular cortisol, into the blood. For the majority of bacterial fish pathogens, stress is considered a key factor in disease outbreaks. Based upon studies in mammals, there is considerable evidence to suggest that, besides impairing the immune system, cortisol can have a direct effect on bacterial cells. Hitherto, this intriguing field of microbial endocrinology has remained largely unexplored in aquatic diseases. The present study investigated in vitro the impact of cortisol on phenotypic traits of the fresh water fish pathogen *Flavobacterium columnare*. Colonies obtained from the highly virulent (HV) isolates resulted in significantly larger and more spreading colonies compared to those from the low virulent (LV) isolates. High cortisol doses added displayed a direct effect on the bacterial cells and induced a significant decrease in colony size. An additional intriguing finding was the inverse relationship between cortisol concentrations added to the broth and the spreading character of colonies retrieved, with higher cortisol doses resulting in less rhizoid to rough and even smooth colony formation (the latter only in the LV trout isolate), suggesting a dose–response effect. The loss of the rhizoid appearance of the *F. columnare* colonies upon administration of cortisol, and hence the loss of motility, might indicate a phenotypic change to the biofilm state. These findings form the basis for further research on the impact of glucocorticoids on other virulence factors and biofilm formation of *F. columnare*.

**Electronic supplementary material:**

The online version of this article (doi:10.1186/s13567-016-0370-9) contains supplementary material, which is available to authorized users.

## Introduction

For the majority of bacterial fish pathogens, stress is considered a key factor in disease outbreaks. Based upon studies in mammalian species, there is considerable evidence to suggest that, besides eliciting an impairment of the immune system, stress hormones can also have a direct effect on the bacterial cells [1, 2]. Hitherto, this intriguing field of microbial endocrinology, whereby micro-organisms, through their long co-existence with animals, have developed sensory systems for detecting host-associated hormones [3], has remained largely unexplored in aquatic diseases.

Teleost fish faced with stressful stimuli launch an endocrine stress response through activation of the hypothalamic-pituitary-interrenal (HPI-) axis to release glucocorticoids, in particular cortisol into the blood [4, 5]. Cortisol elicits a series of physiological and behavioural changes [6–8] that allow the fish to cope with altered situations [9–11].

A positive correlation between cortisol concentrations in plasma and mucus was demonstrated [12, 13]. Mucus hence may contain cortisol via endogenous exposure. Importantly, some fish may be predisposed to consistently exhibit high or low cortisol responses to stressors. Indeed, a considerable level of individual variation in the magnitude of cortisol elevation in response to a 90 min transportation or shallow water housing was noted in carp (*Cyprinus carpio*) [12] and rainbow trout (*Oncorhynchus mykiss*) [14]. Fish may release cortisol through the gills, urine or feces into the surrounding water [15]. In addition to cortisol being present in the mucus via endogenous exposure, it may also be taken up from the water, which is termed exogenous exposure. Thereby, fish housed in the same water body are exposed to cortisol levels which may accumulate especially in recirculation aquaculture systems where water renewal is limited and stocking densities are high [16].

A much-feared and predominant bacteriosis of freshwater fish species is columnaris disease, caused by the gram-negative fish pathogen *Flavobacterium columnare* (*F. columnare*) [17]. This disease causes major financial losses in important aquaculture species such as rainbow trout [18] and carp [19, 20]. However, many knowledge gaps on its pathogenesis remain open. In former in vivo trials in carp, after inoculation with the low virulent isolate, marked variations in response were noted between the fish. While 90–95% of the fish remained clinically healthy throughout the trial, 5–10% of the carp housed in the same tank revealed macro- and microscopic lesions comparable to those observed in the carp inoculated with the highly virulent isolate [21, 22]. We hypothesize that this might be rooted in the most susceptible fish firstly succumbing to columnaris disease, with susceptibility being defined as displaying altered cortisol levels in the plasma and gill mucus. In this line of reasoning, we further launch the hypothesis that cortisol might have a direct impact on the *F. columnare* bacterial cells, influencing the expression of bacterial traits involved in host colonization and/or other virulence-associated determinants.

In this respect, the present study aimed to investigate the impact of cortisol on *F. columnare* in vitro. To do so, firstly, an ultra-performance liquid chromatography coupled to tandem mass spectrometry (UPLC-MS/MS) method for quantifying cortisol in modified Shieh medium [23, 24] was developed and validated. Subsequently, *F. columnare* isolates of carp and trout of differing virulence were cultivated in the presence or absence of cortisol and the impact on bacterial titers and colony morphology assessed.

## Materials and methods

### Development and validation of a UPLC-MS/MS method for cortisol analysis in modified Shieh broth

Chromatographic analysis was performed on an Acquity UPLC-MS/MS Xevo TQS using an Acquity Ultra Performance LC BEH C_18_ (1.7 µm; 2.1 × 100 mm) column (Waters, Milford, USA). Samples were evaporated to dryness with a Turbovap™ nitrogen evaporator (Biotage, Sweden). Grace PureTM SPE C_18_-Max (500 mg, 6 mL) columns for solid-phase extraction (SPE) were obtained from Grace Davison Discovery Sciences (Lokeren, Belgium). High-performance liquid chromatography (HPLC)-gradient grade methanol [Hipersolv Chromanorm, obtained from VWR International BVBA (Leuven, Belgium)] was used as extraction solvent, while methanol absolute LC-MS, formic acid ULC-MS grade [Biosolve BV (Valkenswaard, The Netherlands)] and ultrapure water of a Milli-Q gradient Q-Gard 2 [Millipore (Billerica, USA)] were used as mobile phase solvents. All products used had a certificate of analysis. Cortisol was purchased from Sigma-Aldrich (Diegem, Belgium). Cortisol-d_4_ (purchased from CDN Isotopes (Pointe-Claire, Canada)) was used as an internal standard.

Approximately 1.2 mL of modified Shieh was sampled and filtered over a filter tube (particle retention = 0.2 µm) into a 2 mL Eppendorf tube. Next, the amount of filtered sample used for analysis was standardized at 1 mL and pipetted into a 10 mL test tube. Subsequently, 3990 µL of MilliQ water and 10 µL of a cortisol-d_4_ solution of 0.5 µg/L were added as internal standard. When lower amounts of sample were used, the volume of cortisol-d_4_ was adapted accordingly. The sample was vortex-mixed for 30 s to homogenize. After conditioning a C_18_ SPE column with 3 mL of methanol followed by 3 mL of ultrapure water, the sample was loaded. The column was washed with 4.5 mL H_2_O/MeOH (65:35; v/v) and retained compounds were eluted with 2.5 mL H_2_O/MeOH (20:80; v/v) into a 10 mL test tube and evaporated to dryness under nitrogen at 60 °C using a nitrogen evaporator. The sample was finally reconstituted in 50 µL H_2_O/MeOH (80:20; v/v) in a vial with insert and analyzed by means of UPLC-MS/MS. As reference for future research, matrix-matched calibration curves were set-up in 1 mL of modified Shieh. Calibration standards in the validation study ranged from 0.05 to 50 µg/L. For the analysis of samples from studies dealing with the impact of cortisol on growth characteristics of *F. columnare*, three additional calibration standards were included of 500, 1000 and 5000 µg/L, respectively.

Cortisol was separated from the medium, identified and quantified as described by Aerts et al. [25] and data analysis was performed using Masslynx software from Waters; analysis results were reported as the value (µg/L) ± the expanded measurement uncertainty (U) (µg/L) with a coverage factor (k) of 2 (95% confidentiality interval).

Validation samples were made by aliquotation of one batch of modified Shieh. No certified reference material, inter-laboratory comparison tests or any other validated methods for the above mentioned compound/matrix combinations existed and hence validation was done using standard addition to validation samples. Five concentration levels, ranging from 0.05 to 50 µg/L, were tested in fivefold and this was repeated on four different days within a period of 1 month under intra-reproducibility conditions, i.e. by two persons using different solutions and one UPLC-MS/MS system. All validation experiments were carried out by authorized personnel in a controlled environment with calibrated equipment and controlled solutions according to the requirements of the standard EN ISO/IEC 17025 [26]. Analysis was done using standardized sequences consisting of different calibration standards, blanks, negative and positive controls (all in modified Shieh broth). Results for every compound were evaluated by assessing the (relative) retention time and relative ion intensities of the compound and fragments.

All validation parameters were determined and evaluated according to the requirements of the commission decision No. 657/EC [27]. The apparent recovery (AR) as well as precision (repeatability and intra-laboratory reproducibility) for cortisol were determined under intra-laboratory reproducibility conditions resulting in 20 analyses for every concentration level and a total of 100 analyses per compound. Due to the low concentration levels, a Dixon’s outlier test (ISO/DIS 5725 [28]) and a Grubbs’ test [29] were performed to detect possible outliers. Based on pre-validation analyses, the initial working range for validation was set from 0.05 to 50 µg/L, which was expanded to 5000 µg/L in the framework of the experiment. The linearity of the calibration curve was determined by comparison of the experimental and theoretical curve. The decision limit (CCα) was calculated as the intercept of the calibration curve plus 2.33 times the standard deviation on the intra-laboratory reproducibility (α = 1%), while the detection capability (CCβ) was calculated as the concentration of CCα plus 1.64 times the standard deviation on the intra-laboratory reproducibility (β = 5%). The sensitivity of the method was determined by a dilution experiment on blank matrix samples, while selectivity was tested by analyzing blank and spiked samples (using compounds with similar physical and chemical properties such as tetrahydrocortisol). In addition, the robustness of the method and stability of the compounds in diluent as well as in modified Shieh broth were monitored using trendcharts during method development and subsequent validation. Finally, U was determined by linear summation as well as by quadratic summation or Nordtest method [30] as there is no consensus in the literature on a preferred method.

### The impact of cortisol on *F. columnare* in vitro growth characteristics

Four *F. columnare* isolates (0901393, CDI-A, JIP P11/91 and JIP 44/87) were adopted [17]. Their virulence profile was determined previously [21, 22]. Isolates that were able to elicit 80% mortality or more within 72 h were assigned as highly virulent (HV), whereas isolates causing 20% mortality or less were designated low virulent (LV) [21, 22]. Isolates 0901393 and CDI-A were recovered from carp and proved to be HV and LV, respectively. Isolates JIP P11/91 and JIP 44/87 were obtained from rainbow trout and were assigned as HV and LV, respectively. All four isolates belonged to genomovar I, as determined at the Aquatic Microbiology Laboratory of Auburn University (Alabama, USA) using 16S-restriction fragment length polymorphism according to the protocol described by Olivares–Fuster et al. [31]. The isolates were grown in triplicate for 36 h at 28 °C on modified Shieh agar plates. For each isolate and per plate, five randomly selected colonies were sampled and transferred to 15 mL Falcon tubes filled with 13 mL of modified Shieh broth, which were placed overnight on a shaker at 28 °C at 100 rpm. Two and a half mL of these cultivated broths were added to 22.5 mL of modified Shieh broth in 50 mL Falcon tubes (hence a tenfold dilution). Cortisol was dissolved in ultrapure water, filtered through a 0.2 µm filter (Millipore, Bedford, USA) and diluted to obtain final concentrations in the diluted broth cultures of 5000, 1000 and 500 µg/L accounting for 13.78, 2.76 and 1.38 µM cortisol, respectively. Per isolate, each of these three concentrations and a control broth to which only sterile ultrapure water was added, were tested in triplicate. Using the UPLC-MS/MS method developed in the aforementioned research, the cortisol concentrations were measured at 24 h following the addition of cortisol by collecting subsample volumes of 1.2 mL of cell-free culture supernatans in triplicate. Furthermore, 24 h after adding cortisol, the bacterial titers of all samples were determined using tenfold macrodilution series in triplicate by adding 0.5 mL of the bacterial cultures to 4.5 mL sterile modified Shieh broth. Of each dilution, 50 µL was inoculated on modified Shieh agar plates and incubated for 48 h at 28 °C. Colony morphology was interpreted at the highest dilution at which growth occurred. The colonies were classified as rhizoid, rough or smooth according to Kunttu et al. [32, 33]. Rhizoid colonies have spreading tendrils radiating from a denser center, rough colonies have irregularly shaped dense colony centers with frayed edges, and smooth colonies have irregularly to round shaped colonies with smooth edges. The colonies retrieved from the highest dilution were photographed using a stereomicroscope (Olympus SZX7 with a color view I camera of Soft Imaging System). Per plate, the mean diameter of the bacterial colonies and the mean length of the radiating tendrils were determined for three randomly selected photographed colonies. The mean length of radiating tendrils was used as a reflection of the spreading character of the colony. The mean out of three measured diameters per colony and the mean length out of three radiating tendrils were calculated using microscope and software tools of the Olympus SZX7.

All parameters were statistically modeled using linear mixed models (proc GLIMMIX) in SAS 9.4 (SAS Institute Inc., Cary, NC, USA) with cortisol concentration, virulence of the isolate, fish species, and their interaction as fixed effects. Non-significant interactions were removed from the final models. All other possible interactions were always non-significant and therefore removed from the final models. Results of both fish species were gathered in one statistical model in order to increase power and to observe possible conserved effects over species. A random effect for plate was introduced to correct for repeated measures within plates. The analysed data were considered normally distributed, based on the graphical evaluation (histogram and QQ-plot) of the residuals. Bacterial titers were log10-transformed to obtain normally distributed residuals. A post hoc Tukey–Kramer test was performed to compare treatments with controls. Statistical results were considered to be significant when *p*-values were lower than 0.05. A *p*-value between 0.05 and 0.1 was considered as a trend.

## Results

### Development and validation of a UPLC-MS/MS method for cortisol analysis in modified Shieh broth

All validation parameters were determined and evaluated according the requirements of the Commission Decision No. 657/EC [27]. When assessing the linearity for cortisol in the defined working range, a value of the correlation coefficient R^2^ ≥ 0.995 (R^2^ = 0.9998) was found. Furthermore, the residuals of the calculated model for cortisol were normally distributed. CCα and CCβ were 0.011 and 0.017 µg/L, respectively. Table 1 shows the results for trueness (apparent recovery —AR), precision (variation coefficient—VC) and the expanded measurement uncertainty (U) (µg/L) for cortisol analysis in modified Shieh samples. Finally, the method showed to be highly sensitive and specific and was declared to be “fit for purpose”.

**Table 1:** **Results for trueness, precision and the expanded measurement uncertainty for cortisol analysis in modified Shieh**

Cortisol level (µg/L)	Outliers (n)	Values (n)	AR (%)	VC_r_ (%)	VC_R_ (%)	U^a^ (k = 2) (%)	U^a ^(k = 3) (%)	U^b ^(k = 2) (%)
0.05	1	19	104.21	8.01	9.62	23.45	33.07	45.17
0.25	0	20	95.18	2.94	2.91	10.64	13.55	36.86
1.00	0	20	99.10	1.92	2.85	6.60	9.45	35.60
5.00	0	20	99.40	1.76	1.89	4.38	6.27	35.07
50.00	0	20	98.79	1.74	2.20	5.61	7.81	35.27

AR: apparent recovery, VC_r_: variation coefficient (repeatability), VC_R_: variation coefficient (reproducibility), U: expanded measurement uncertainty.

^a^Calculation of U using linear summation with k = 2 (95% confidentiality interval) and k = 3 (99% confidentiality interval).

^b^Calculation of U using quadratic summation with k = 2 (95% confidentiality interval).

### The impact of cortisol on *F. columnare* in vitro growth characteristics

#### Bacterial titers

**Figure 1:**
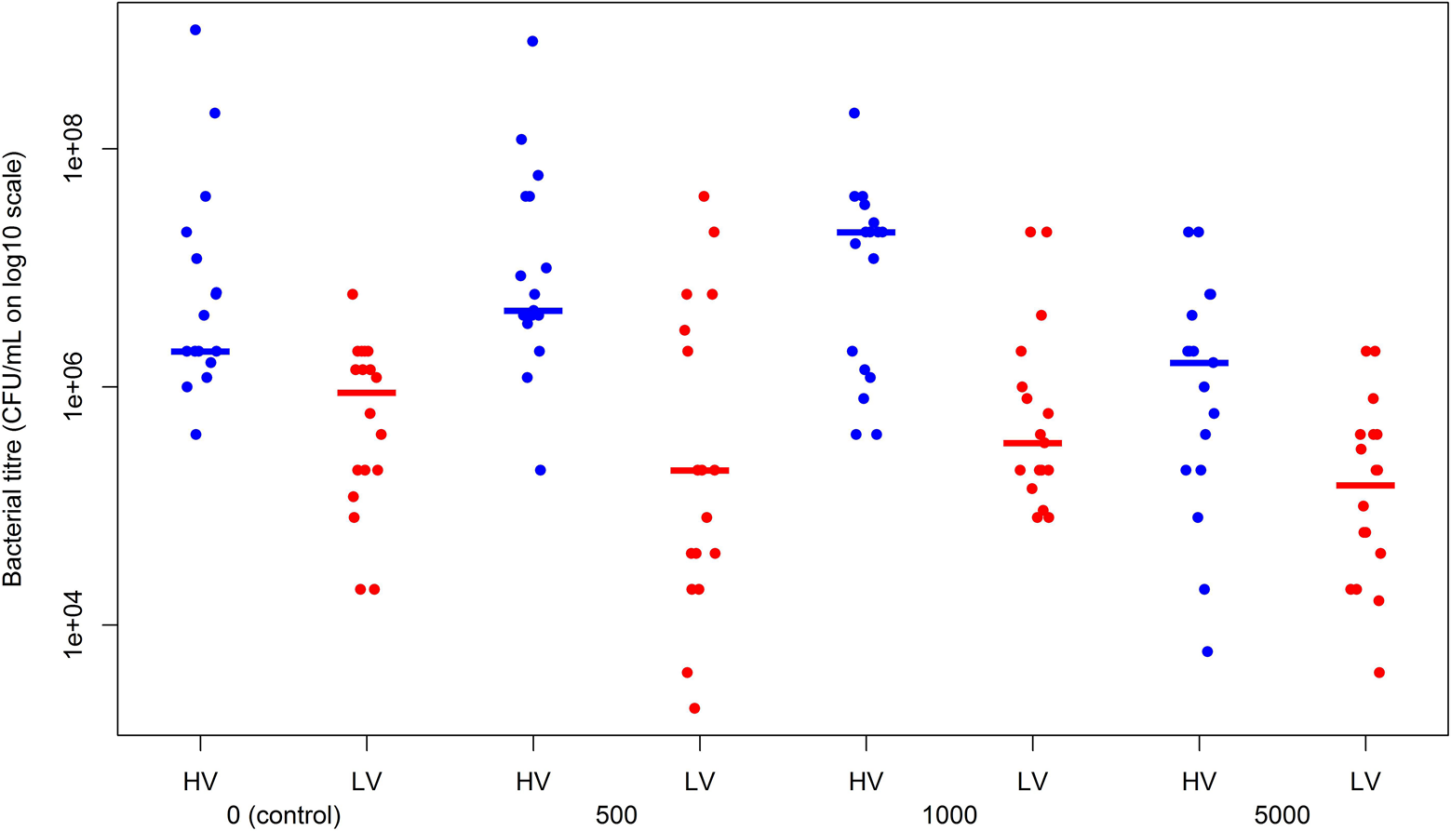
**Bacterial cell counts.** The Y-axis represents the log10 transformed bacterial cell counts (log10 CFU/mL) retrieved from the highly (HV) and low virulent (LV) isolate inoculated modified Shieh broths 24 h after addition of the appropriate cortisol concentrations (µg/L). The latter are displayed on the X-axis. The blue dots represent the bacterial cell counts retrieved from the broths of the HV isolates, the red dots those from the broths of the LV isolates. Averaged values per cortisol concentration and per virulence group are indicated by the flat colored lines.

The bacterial cell counts (Figure 1) were significantly influenced by the cortisol concentration added to the inoculated broths (*p* = 0.047) (Table 2). Upon comparing different treatments, a decreasing trend (*p* = 0.053) in bacterial titers was found for broths to which 5000 vs. 1000 µg/L cortisol was added. Indeed, bacterial titers retrieved from broths to which 1000 µg/L was added, were 0.84 ± 0.33 times higher compared to the ones to which 5000 µg/L was added. Other bacterial titers retrieved also resulted in numerically higher but not statistically significant bacterial cell counts upon comparing them with the bacterial cell counts retrieved from the broths to which 5000 µg/L was added (Table 2).

**Table 2:** **Bacterial cell counts (BCC) and bacterial colony sizes (BCS)**

	Added cortisol concentration (µg/L)				*p*-value
	0 (control)	500	1000	5000	
BCC (log10 CFU/mL)	6.21 ± 0.23	6.13 ± 0.23	6.32 ± 0.23	5.48 ± 0.23	0.047
BCS (µm/mL)	3421 ± 178	2765 ± 178	3128 ± 178	3421 ± 178	0.014

Least Squares Means and standard errors (LSM ± SE) of the log10 transformed bacterial cell counts (BCC) (log10 CFU/mL) and bacterial colony sizes (BCS) (µm) retrieved from the highly and low virulent isolates inoculated modified Shieh broths 24 h after addition of the appropriate cortisol concentrations [cortisol] (µg/L).

The average bacterial cell titers differed significantly between both virulence groups (*p* < 0.001). The average bacterial titers retrieved from the HV versus LV isolate inoculated broths were 6.60 ± 0.16 and 5.47 ± 0.16 log10 CFU/mL, respectively. Hence, the average bacterial titer retrieved from the HV isolate inoculated broth was 11.26 ± 0.23 times higher compared to the average titer retrieved from the LV isolate inoculated broth (*p* < 0.001).

#### Colony morphology

The colonies retrieved from the control broth inoculated with the HV isolates were classified as rhizoid. For the cortisol supplemented broth, the colonies displayed a slightly rhizoid going to rough morphology with the rhizoid characteristics being less pronounced as the cortisol concentration increased. The appearance of the colonies originating from the control and cortisol supplemented broths of the LV carp isolate was rhizoid and slightly rhizoid to rough, respectively. For the trout, the morphology of colonies retrieved from the LV isolate inoculated control broth was slightly rhizoid, rough or smooth, while it was rough or smooth when cortisol was supplemented. These results are depicted in Additional file 1.

##### Colony size

**Figure 2:**
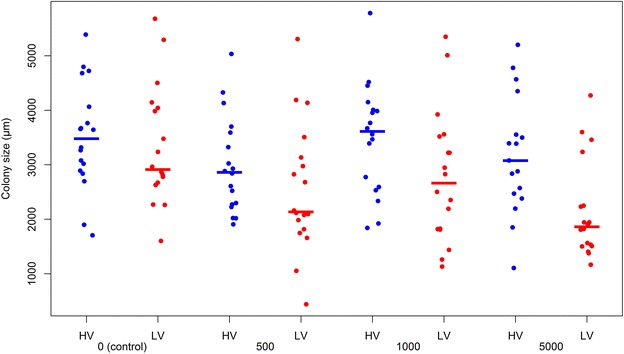
**Bacterial colony sizes.** The Y-axis represents the bacterial colony sizes in µm retrieved from colonies of the highly (HV) and low virulent (LV) isolate inoculated modified Shieh broths 24 h after addition of the appropriate cortisol concentrations (µg/L). The latter are displayed on the X-axis. The blue dots represent the bacterial cell counts retrieved from the broths of the HV isolates, the red dots those from the broths of the LV isolates. Averaged values per cortisol concentration and per virulence group are indicated by the colored lines.

The bacterial colony size (Figure 2) was significantly influenced by the cortisol dose added (*p* = 0.014) (Table 2). Addition of 5000 µg/L of cortisol to the inoculated broths, resulted in 753 ± 253 µm (adj *p* = 0.019) significantly smaller bacterial colony sizes compared to colonies measured from the unsupplemented control broths.

Furthermore, the average bacterial colony sizes differed significantly between both virulence groups (*p* = 0.001). The average bacterial colony sizes retrieved from the HV and LV isolate inoculated broths of carp and trout were 3291 ± 127 and 2700 ± 126 µm, respectively. Hence, the average bacterial colony sizes retrieved from the HV isolate inoculated broths were 591 ± 179 µm larger compared to the colony measurements retrieved from the LV isolate inoculated broths (*p* = 0.001).

Fish species also influenced the average bacterial colony size significantly (*p* < 0.001). Colony sizes retrieved from the carp isolates were on average 735 ± 178 µm larger compared to those from the trout isolates (*p* = 0.001).

##### Colony spreading

**Figure 3:**
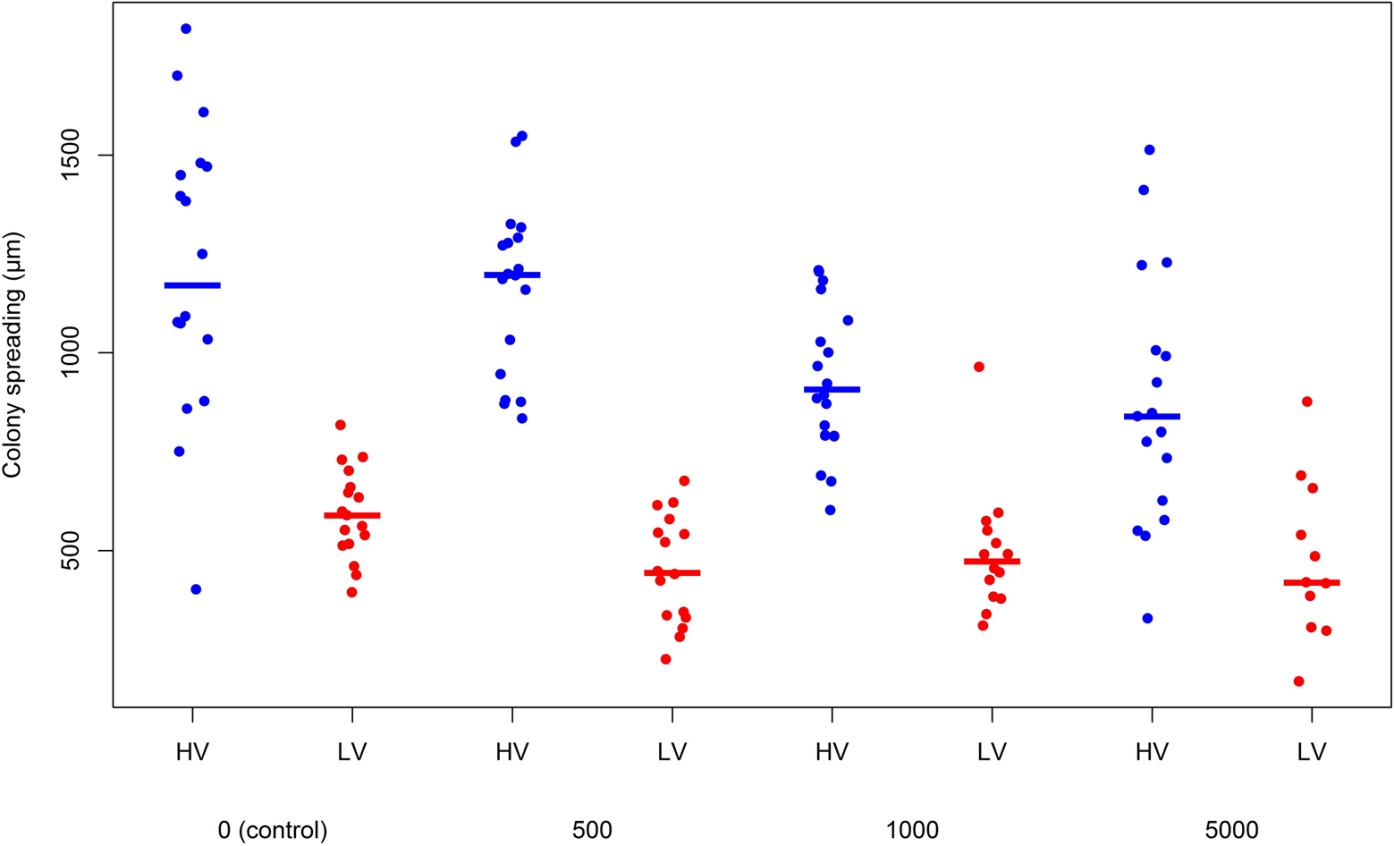
**Bacterial colony spreading.** The Y-axis represents the bacterial colony spreading in µm retrieved from the colonies of the highly (HV) and low virulent (LV) isolate inoculated modified Shieh broths 24 h after addition of the appropriate cortisol concentrations (µg/L). The latter are displayed on the X-axis. The blue dots represent the bacterial cell counts retrieved from the broths of the HV isolates, the red dots those from the broths of the LV isolates. Averaged values per cortisol concentration and per virulence group are indicated by the colored lines.

The bacterial colony spreading (Figure 3) was significantly influenced by the cortisol dose (*p* = 0.002) and the virulence group (*p* < 0.001) (Table 3). A significant interaction between these both parameters was noted (*p* = 0.041).

**Table 3:** **Bacterial colony spreading (BCSp)**

	Added cortisol concentration (µg/L)			
	0 (control)	500	1000	5000
BCSp HV (µm/mL)	1211 ± 58^a^	1164 ± 58^a^	932 ± 58^b^	876 ± 60^b^
BCSp LV (µm/mL)	593 ± 60^a^	452 ± 61^a^	496 ± 65^a^	479 ± 73^a^
*p*-value within [cortisol]	<0.001	<0.001	<0.001	<0.001

Least Squares Means and standard errors (LSM ± SE) of the bacterial colony spreading (BCSp) (µm) measured from the colonies of the highly (HV) and low virulent (LV) isolates inoculated modified Shieh broths 24 h after addition of the appropriate cortisol concentrations [cortisol] (µg/L).

^a,b^Refer to significant differences within one virulence group (HV or LV).

No statistically significant differences were observed for the average bacterial colony spreading in the LV isolate inoculated broths (*p* = 0.390). However, bacterial colony spreading was significantly influenced in the HV isolate inoculated broths (*p* < 0.001). Upon adding 1000 or 5000 µg/L cortisol to the HV isolate inoculated broths, less spreading was measured upon comparison with the unsupplemented HV isolate broths and the HV isolate inoculated broths to which 500 µg/L cortisol was added (all adj *p* < 0.05) (Table 3).

Upon comparing average colony spreading results obtained from HV versus LV isolate supplemented broths within each corresponding cortisol treatment, the colony spreading measured from the HV isolates was significantly higher (all adj *p* < 0.001) than that from the LV isolate inoculated broths, as presented in Table 3.

## Discussion

The response to stress is considered “*an adaptive mechanism that allows fish to cope with real or perceived stressors in order to maintain its normal or homeostatic state*” [4]. When fish encounter stress, the body will try to re-establish that dynamic equilibrium by a suite of adaptive responses. In case the healthy steady state cannot be attained, pathology may ensue [34], which is an important concern for health care managers and aquaculturists.

The stress hormone cortisol is recognized as the principle glucocorticoid released in teleostean fish [4]. Fish can react very diverse to stress based on genetic (e.g. species, strain), developmental (e.g. life history stage), and environmental (e.g. temperature, nutrition, water quality) traits [4]. Due to this confirmed fish species-specificity in response to stress [12, 14], this study included the effect of cortisol on bacterial isolates retrieved from both carp and trout.

As a method for quantitation of cortisol in modified Shieh broth was lacking in the literature, a UPLC-MS/MS method was developed. This was done in an EN ISO/IEC 17025 [26] regulated environment. Subsequent methodological validation was set according the requirements of the Commission Decision No. 2002/657/EC [27]. An initial working range from 0.05 to 50 µg/L, enabling to detect extremely low concentrations, was chosen. In a next step, the working range was expanded to 5000 µg/L allowing to quantify cortisol at much higher concentrations [12, 14].

The circulating level of cortisol is commonly used as an indicator of the degree of stress experienced by fish [5, 35]. Plasma cortisol levels encountered in carp and trout blood following an acute stressor resulted in average values of 400 ± 190 and 140 ± 5 µg/L, respectively [12]. These values correspond to the lowest cortisol concentration of 500 µg/L used in this study. In chub (*Leuciscus cephalus*), mean blood cortisol levels during stress even reached 1500 µg/L [14], leaning more towards the two higher cortisol levels of 1000 and 5000 µg/L chosen in the present in vitro trials. It however remains unclear whether blood or mucus cortisol levels of 5000 µg/L could be reached during an infection with columnaris disease and hence deserves further investigation.

Our former histopathological and ultrastructural examination of carp and rainbow trout inoculated with a HV *F. columnare* isolate, disclosed bacterial biofilm formation and concomitant destruction of the gill tissue, allowing direct contact between the bacterial cells and the blood [22]. A biofilm is a “*microbially derived sessile community characterized by cells that are irreversibly attached to a substratum or interface or to each other, are embedded in a matrix of extracellular polymeric substances that they have produced, and exhibit an altered phenotype with respect to growth rate and gene transcription*” [36]. Biofilm development requires several key steps. Sauer et al. [37] characterized five stages of biofilm development in *Pseudomonas aeruginosa*: (i) reversible attachment, (ii) irreversible attachment, (iii) a first maturation stage, (iv) a second maturation stage, and (v) dispersion. Irreversible attachment occurs when the bacterial cells start clustering. In this phase, attached cells lose motility [37] and exhibit a changed phenotype compared to planktonic cells [36, 38]. This process was also described for *F. columnare* [39].

Bacterial motility is an important factor for rapid colonization of a surface [40]. The motility rate has been linked to changes in the expression of virulence factors in different pathogenic Gram-negative bacteria [41–45]. In *F. columnare* as well, the gliding motility is a well-known characteristic [39, 46–48]. The high motility of *F. columnare* bacterial cells is reflected by the rhizoid shape of the harboring colonies, a feature which in itself has been positively linked to virulence [33]. In the current study, colonies obtained from the HV isolates indeed were significantly larger and more spreading compared to those from the LV isolates, both with and without the addition of cortisol. Biofilm formation on the fish’s skin and gills is a well-known feature in fish succumbing to columnaris disease. The formation of biofilms is particularly relevant for immunocompromised fish, lacking the ability to counter invading organisms [36]. Fish may react differently in behavioral responses to certain stress situations [49]. In our former in vivo research we observed that the HV isolate succeeded in colonizing the complete gill tissue in 100% of the fish, while for the LV isolate the gills were more focally colonized and this in only 5–10% of the inoculated animals [21, 22]. As has been suggested for other microorganisms [2, 50, 51], *F. columnare* could have developed sensory systems for detecting the host-associated cortisol and possible other glucocorticoids.

Cortisol did display a direct effect on the bacterial cells. Cortisol induced a significant decrease in colony size. The colonies procured from broth to which 5000 µg/L cortisol was added, were significantly smaller compared to those from the unsupplemented control broth. An additional intriguing finding was the inverse relationship between cortisol concentrations added to the broth and the spreading character of colonies retrieved, with higher cortisol doses resulting in less rhizoid to rough and even smooth colony formation (the latter only in the LV trout isolate), suggesting a dose–response effect. Indeed, the colonies retrieved from the control broths or the HV isolate inoculated broths to which 500 µg/L cortisol was added, demonstrated significantly more spreading compared to those retrieved from the HV isolate broths to which 1000 or 5000 µg/L was added. Loss of gliding motility in *F. columnare* was suggested to appear as non-rhizoid colony morphology [32]. In this study, the loss of the rhizoid appearance of the *F. columnare* colonies upon administration of cortisol, and hence the loss of motility, might indicate a phenotypic change into the biofilm state.

No significant differences were found upon comparing different groups for bacterial cell counts upon addition of cortisol. We can hence not conclude from these results that cortisol could significantly impact planktonic growth. For future research, it would also be interesting to assess not only bacterial growth characteristics of the planktonic cells, but to furthermore include assays which determine bacterial cell counts of sessile cells.

In summary, this in vitro study demonstrates a direct impact of cortisol on several phenotypical growth characteristics, such as bacterial colony size and colony spreading and hence gliding ability of the fish pathogenic bacterium, *F. columnare*. This line of reasoning engenders a new perspective to bacteria-host communications in aquaculture. These findings form the basis for further research on the impact of glucocorticoids on other virulence factors and biofilm formation of *F. columnare*. Indeed, the elucidation of the mechanisms through which stress and the release of glucocorticoids alter susceptibility to columnaris disease in fish could help improve its prevention and treatment.

## Additional file

10.1186/s13567-016-0370-9 **Stereomicroscopic appearance of bacterial colonies procured from the control and cortisol supplemented modified Shieh broth.** Cortisol concentrations applied were 500, 1000, 5000 µg/L for the highly virulent and low virulent carp and trout *F. columnare* isolates. Rhizoid colonies have spreading tendrils radiating from a denser center, rough colonies have irregularly shaped dense colony centers with frayed edges, and smooth colonies have irregularly to round shaped colonies with smooth edges.
