# Supplementary material for: Cortisol directly impacts Flavobacterium columnare in vitro growth characteristics
Source: Vet Res. 2016 Aug 17;47:84. doi: 10.1186/s13567-016-0370-9 (PMC4987970; doi:10.1186/s13567-016-0370-9)
Supplement: Supplementary file 1 — 10.1186/s13567-016-0370-9 Stereomicroscopic appearance of bacterial colonies procured from the control and cortisol supplemented modified Shieh broth. Cortisol concentrations applied were 500, 1000, 5000 µg/L for the highly virulent and low virulent carp and trout F. columnare isolates. Rhizoid colonies have spreading tendrils radiating from a denser center, rough colonies have irregularly shaped dense colony centers with frayed edges, and smooth colonies have irregularly to round shaped colonies with smooth edges. [file 13567_2016_370_MOESM1_ESM.docx]

**Additional file 1 Stereomicroscopic appearance of bacterial colonies procured from the control and cortisol supplemented modified Shieh broth.**

| **Isolate** | **Control** | **Cortisol [500 µg/L]** | **Cortisol [1000 µg/L]** | **Cortisol [5000 µg/L]** |
| --- | --- | --- | --- | --- |
| **Low virulent koi carp isolate CDI-A** | Rhizoid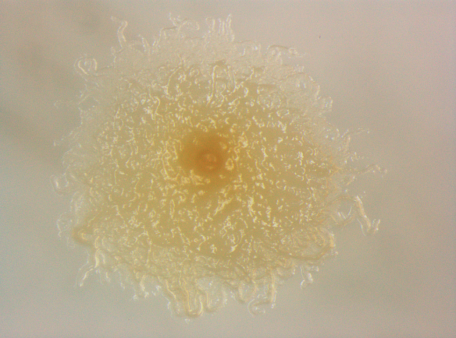 | Slightly rhizoid  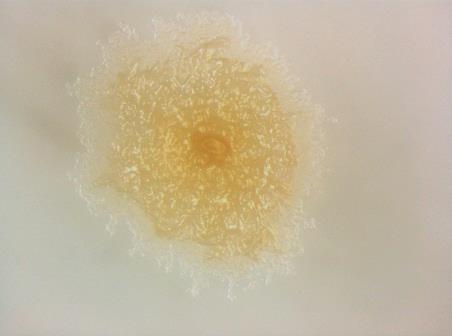 | Slightly rhizoid to rough  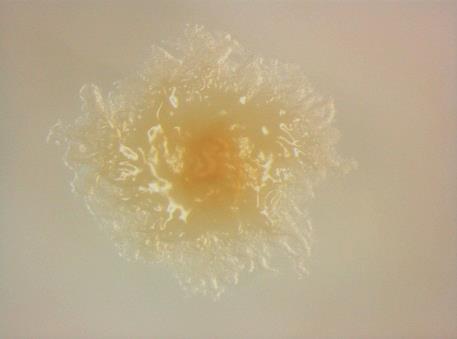 | Rough  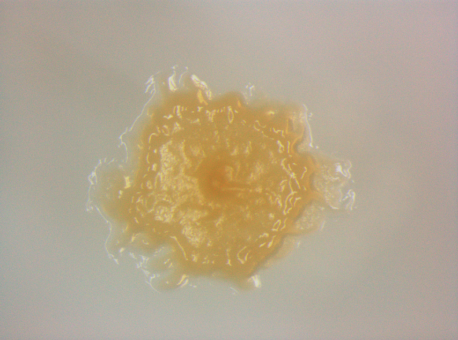 |
| **Highly virulent koi carp isolate 0901393** | Rhizoid  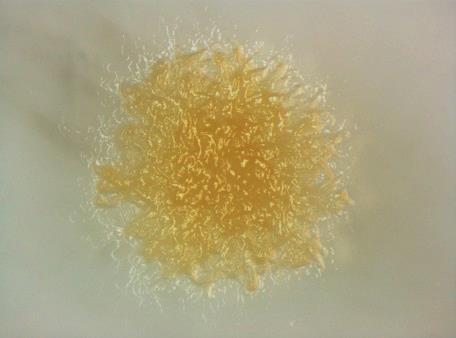 | Rhizoid  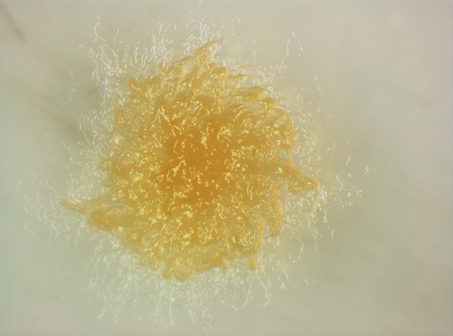 | Rhizoid  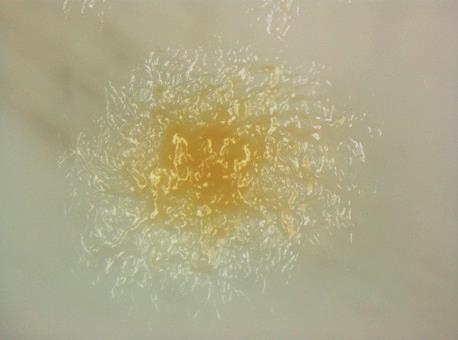 | Slightly rhizoid to rough  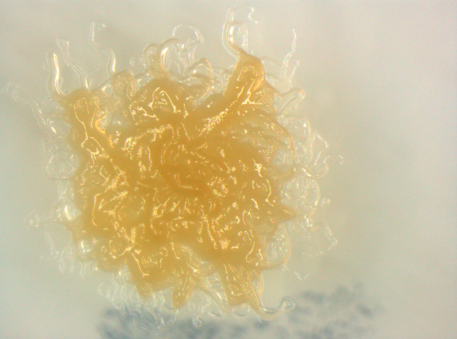 |
| **Low virulent trout isolate JIP 44/87** | Rough  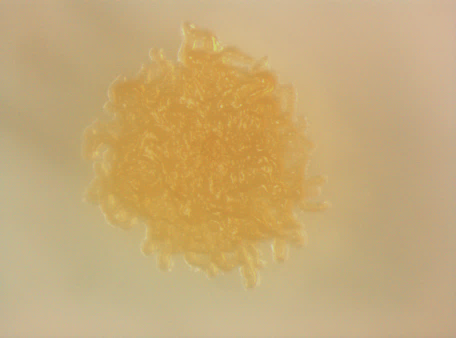 | Rough  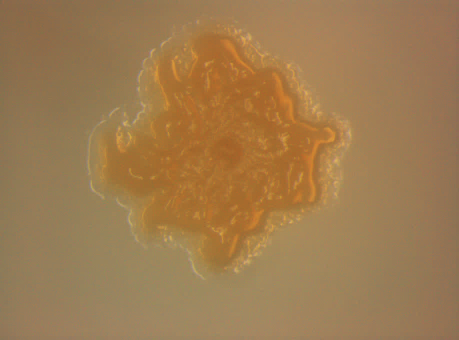 | Smooth  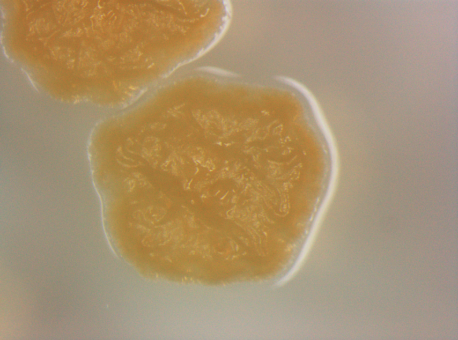 | Smooth  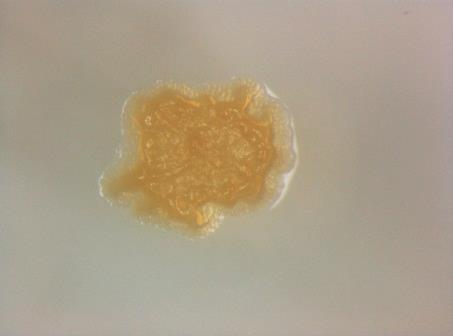 |
| **Highly virulent trout isolate JIP P11/91** | Rhizoid  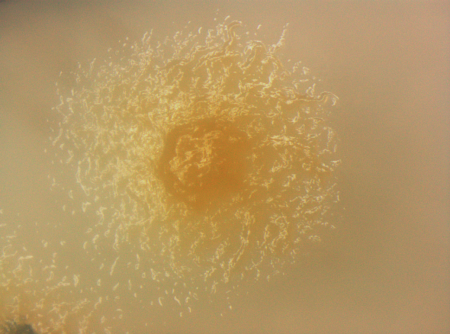 | Rhizoid  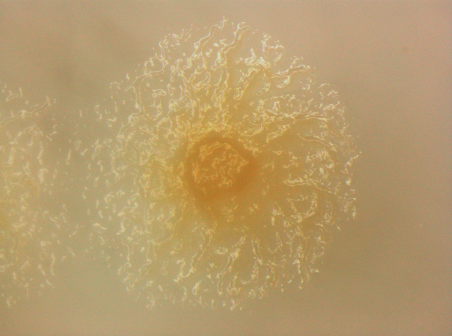 | Slightly rhizoid  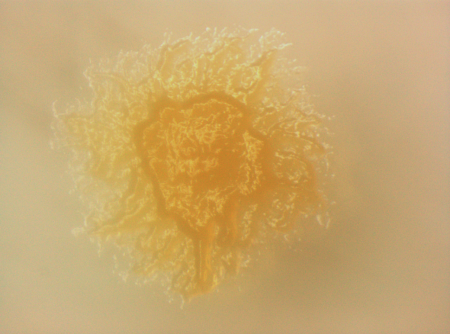 | Rough  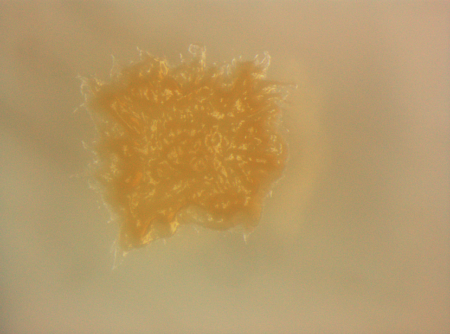 |
|  | | | | |

Cortisol concentrations applied were 0 (control), 500, 1000 and 5000 µg/L for the highly and low virulent carp and trout *F. columnare* isolates. Rhizoid colonies have spreading tendrils radiating from a denser centre, rough colonies have irregularly shaped dense colony centres with frayed edges, and smooth colonies have irregularly to round shaped colonies with smooth edges.
